# Fractional Nadeem trigonometric non-Newtonian (NTNN) fluid model based on Caputo-Fabrizio fractional derivative with heated boundaries

**DOI:** 10.1038/s41598-023-48122-4

**Published:** 2023-12-06

**Authors:** Sohail Nadeem, Bushra Ishtiaq, Jehad Alzabut, Ahmad M. Hassan

**Affiliations:** 1 Department of Mathematics, Quaid-I-Azam University, 45320, Islamabad, 44000 Pakistan; 2 Department of Mathematics and Sciences, Prince Sultan University, 11586 Riyadh, Saudi Arabia; 3 Department of Industrial Engineering, OSTIM Technical University, Ankara, 06374 Turkey; 4 Faculty of Engineering, Future University in Egypt, New Cairo, 11835 Egypt

**Keywords:** Engineering, Mathematics and computing, Nanoscience and technology

## Abstract

The fractional operator of Caputo-Fabrizio has significant advantages in various physical flow problems due to the implementations in manufacturing and engineering fields such as viscoelastic damping in polymer, image processing, wave propagation, and dielectric polymerization. The current study has the main objective of implementation of Caputo-Fabrizio fractional derivative on the flow phenomenon and heat transfer mechanism of trigonometric non-Newtonian fluid. The time-dependent flow mechanism is assumed to be developed through a vertical infinite plate. The thermal radiation’s effects are incorporated into the analysis of heat transfer. With the help of mathematical formulations, the physical flow system is expressed. The governing equations of the flow system acquire the dimensionless form through the involvement of the dimensionless variables. The application of Caputo-Fabrizio derivative is implemented to achieve the fractional model of the dimensionless system. An exact solution of the fractional-based dimensionless system of the equations is acquired through the technique of the Laplace transform. Physical interpretation of temperature and velocity distributions relative to the pertinent parameters is visualized via graphs. The current study concludes that the velocity distributions exhibit an accelerating nature corresponding to the increasing order of the fractional operator. Moreover, the graphical results are more significant corresponding to the greater time period.

## Introduction

Fractional calculus deals with the study of differentiation of fractional order. The real data through a fractional derivative approach can be estimated more effectively as compared to an ordinary derivative. Many fields incorporate the concept of derivatives with fractional order. Examples of such disciplines are fluid dynamics, electrochemistry, dynamic complex systems, and biological systems^1^. The most used fractional derivatives are the Riemann–Liouville derivative having non-integer order and the Caputo derivative. The Riemann–Liouville derivative includes the singular kernel with the non-zero derivative of a constant. The concept of the Caputo derivative has overcome the problem of the Riemann–Liouville derivative by utilizing the power law kernel, which is still singular, but a constant has zero derivative^2^. After that, the concept of Caputo-Fabrizio fractional derivative involves a non-singular kernel of exponential form. With the implementation of numerous models of fractional derivatives, various non-Newtonian fluid models have been examined. An exploration of the fractional derivative on a time-dependent chemically reactive flow behavior of a non-Newtonian fluid in one direction was investigated by Siddique and Bukhari^3^. The authors compared the flow characteristics regarding classical and fractional derivatives and identified significant outcomes in the case of the fractional approach. Antonio Taneco‐Hernández et al.^4^ discussed the time-dependent flow mechanism through a bar by implementing different fractional derivatives. The authors scrutinized the viscoelastic properties of the considered fluid in the context of the various fractional operators. Nadeem et al.^5^ discussed the time-dependent flow of a trigonometric non-Newtonian fluid by considering two heated surfaces. The authors observed that an increase in the fluid parameter results in a reduction of the fluid flow mechanism. Recently many researchers discussed flow problems by adopting the technique of fractional derivatives^6–11^.

In industries and engineering fields, non-Newtonian fluids have various practices. The implementations of such fluids include spray coating, production of paper, lubricants, gaseous diffusions, polymer production, etc. Non-Newtonian fluids have complicated nature which can be examined through numerous mathematical models. With the help of such models, numerous investigations on the behavior of non-Newtonian fluids have been conducted. Abbas et al.^12^ assumed a Riga surface to observe the steady flow nature of a non-Newtonian fluid with physical boundary conditions. The authors explained the characteristics of the flow phenomenon relative to the emerging parameters. Nadeem et al.^13^ studied the radiative flow phenomenon of a non-Newtonian fluid in the context of a stretchable Riga surface. Amplification in the flow field related to improved fluid parameter was identified in their observations. Yang et al.^14^ discussed the model of a non-Newtonian fluid with the involvement of nanoparticles and explored the hydromagnetic flow mechanism on both shrinking and stretching surfaces. The significance of an inclined magnetic field in the flow behavior of a non-Newtonian fluid through a vertical surface was demonstrated by Ishtiaq and Nadeem^15^. An augmentation in the velocity distribution was indicated in their observations corresponding to the improved fluid parameter. Under the influence of physical impacts, the non-Newtonian fluid model with variable characteristics was demonstrated by Ahmad et al.^16^. Through a Riga plate, Nadeem et al.^17^ study a non-Newtonian fluid with its axisymmetric flow nature influenced by variable properties. The authors investigated the flow properties affected by the fluid parameter. The flow characteristics of a non-Newtonian fluid and the impacts of double diffusion theory on the mechanism of heat transport were deliberated by Irfan et al.^18^. The authors numerically and analytically explore the flow phenomenon in relation to significant parameters. The two-phase model-based thermal analysis of a fluid incorporating nanoparticles with the involvement of a movable wedge was inspected by Baby et al.^19^. The study’s findings revealed that an improved wedge parameter led to a diminished fluid flow behavior. Recent studies on the flow of non-Newtonian fluids are exhibited in Refs.^20–24^

The current study has a significant aspect of implementing the Caputo-Fabrizio fractional derivative on the unsteady flow of a non-Newtonian fluid with trigonometric form. No one examined the fractional model of the trigonometric non-Newtonian fluid flow until now. The novelty of the ongoing study includes the fractional-based unsteady flow mechanism of trigonometric non-Newtonian fluid proceed by a vertical infinite plate. The energy equation is exhibited by incorporating the impact of the thermal radiation which is further analyzed with the momentum equation by adopting the definition of the fractional derivative. To acquire an exact solution to the concerned problem, an effective Laplace transform methodology is accomplished. The flow velocity and temperature influenced by numerous pertinent parameters are graphically explained. The purpose of the current study is to address the following queries.

- What are the advantages of using a fractional derivative approach on non-Newtonian fluid flow instead of an ordinary derivative approach?
- How fractional derivative-based equations tackled through the Laplace transform technique?
- What is the impact of the fractional parameter on the boundary layer thickness?
- What effect does the augmented fractional parameter have on the mechanism of heat transport?

## Research methodology

**Figure 1:**
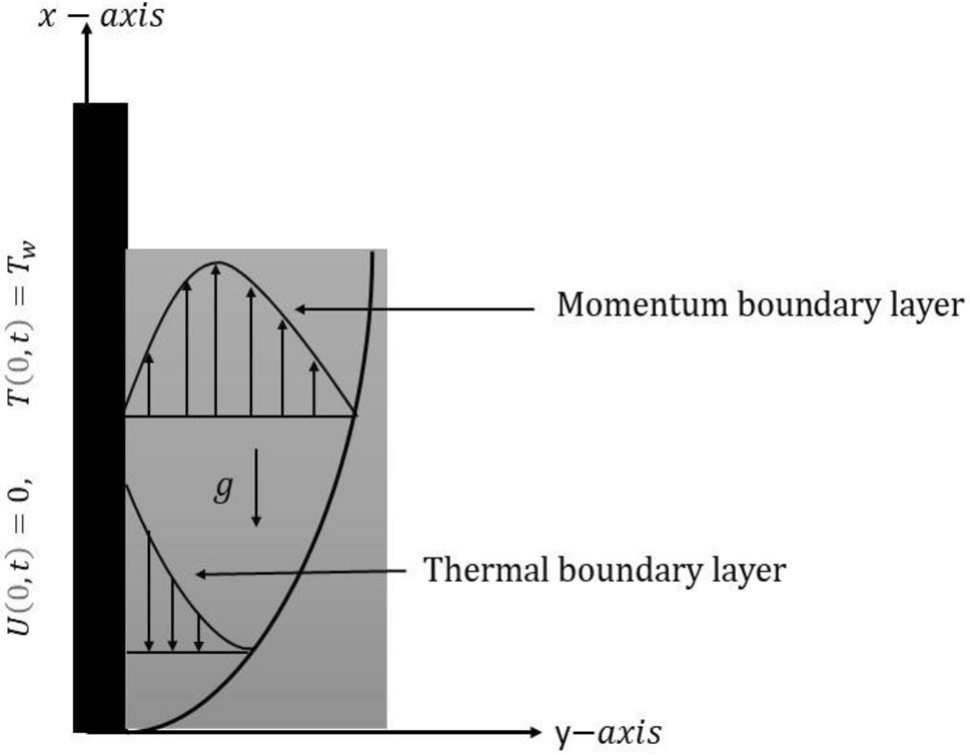
Physical system of problem.

We consider the unsteady flow phenomenon of a non-Newtonian fluid with trigonometric type over a vertical infinite plate. In the setup of Cartesian coordinates, the static plate is placed toward the x-axis and in the perpendicular position of the plate, the y-axis is taken. Initially, both the plate and the considered fluid are in a static position with the fluid’s uniform temperature ${T}_{\infty }$. As the plate is taken to be static, at time $t={0}^{+}$, the plate remains still at rest, but its temperature rises to a constant temperature ${T}_{w}.$ Furthermore, the heat transport mechanism includes the impact of thermal radiation. Figure 1 is prepared for the physical interpretation of the problem.

### Nadeem trigonometric non-Newtonian (NTNN) fluid model

The constitutive equation for the Nadeem trigonometric non-Newtonian (NTNN) fluid model is represented by the following expressions^5^,

1 $${\varvec{\tau}}=-p{\varvec{I}}+{\varvec{S}},$$

In Eq. (1), ${\varvec{S}}$ exhibits the extra stress which is defined in the following way^25^

2 $${\varvec{S}}=\mu {\varvec{A}}+\frac{1}{{\lambda }_{1}\gamma }{\mathrm{sin}h}^{-1}\left(\frac{1}{{\lambda }_{2}}\gamma \right){\varvec{A}},$$

Equation (2) converts into the following form after the first order expansion of ${\mathrm{sin}h}^{-1}\left(\frac{1}{{\lambda }_{2}}\gamma \right)\approx \frac{1}{{\lambda }_{2}}\gamma$.

3 $${\varvec{S}}=\mu {\varvec{A}}+\frac{1}{{\lambda }_{1}{\lambda }_{2}}{\varvec{A}},$$

The expression of Eq. (3) from Eq. (2) can also be obtained if we use ${\mathrm{sin}}^{-1}\left(\frac{1}{{\lambda }_{2}}\gamma \right)$, $\mathrm{sin}\left(\frac{1}{{\lambda }_{2}}\gamma \right)$, ${\mathrm{tan}}^{-1}\left(\frac{1}{{\lambda }_{2}}\gamma \right)$, $\mathrm{tan}h\left(\frac{1}{{\lambda }_{2}}\gamma \right)$, $\mathrm{sin}h\left(\frac{1}{{\lambda }_{2}}\gamma \right)$, and ${\mathrm{tan}h}^{-1}\left(\frac{1}{{\lambda }_{2}}\gamma \right)$ instead of using ${\mathrm{sin}h}^{-1}\left(\frac{1}{{\lambda }_{2}}\gamma \right)$**.** With the help of these trigonometric functions, the extra stress can be written as follows,

4 $$ \begin{gathered} S = \mu A + \frac{1}{{\lambda_{1} \gamma }}\sinh^{ - 1} \left( {\frac{1}{{\lambda_{2} }}\gamma } \right)A, \hfill \\ S = \mu A + \frac{1}{{\lambda_{1} \gamma }}\tanh \left( {\frac{1}{{\lambda_{2} }}\gamma } \right)A, \hfill \\ S = \mu A + \frac{1}{{\lambda_{1} \gamma }}\tan^{ - 1} \left( {\frac{1}{{\lambda_{2} }}\gamma } \right)A, \hfill \\ S = \mu A + \frac{1}{{\lambda_{1} \gamma }}\sin \left( {\frac{1}{{\lambda_{2} }}\gamma } \right)A, \hfill \\ S = \mu A + \frac{1}{{\lambda_{1} \gamma }}\sin^{ - 1} \left( {\frac{1}{{\lambda_{2} }}\gamma } \right)A, \hfill \\ S = \mu A + \frac{1}{{\lambda_{1} \gamma }}\sinh \left( {\frac{1}{{\lambda_{2} }}\gamma } \right)A, \hfill \\ S = \mu A + \frac{1}{{\lambda_{1} \gamma }}\tanh^{ - 1} \left( {\frac{1}{{\lambda_{2} }}\gamma } \right)A, \hfill \\ \end{gathered} $$

By taking the first-order expansion of the above trigonometric functions, we have the following result

$${\mathrm{sin}h}^{-1}\left(\frac{1}{{\lambda }_{2}}\gamma \right)\approx \frac{1}{{\lambda }_{2}}\gamma ,$$

$$\mathrm{tan}h\left(\frac{1}{{\lambda }_{2}}\gamma \right)\approx \frac{1}{{\lambda }_{2}}\gamma ,$$

$${\mathrm{tan}}^{-1}\left(\frac{1}{{\lambda }_{2}}\gamma \right)\approx \frac{1}{{\lambda }_{2}}\gamma ,$$

$$\mathrm{sin}\left(\frac{1}{{\lambda }_{2}}\gamma \right)\approx \frac{1}{{\lambda }_{2}}\gamma ,$$

$${\mathrm{sin}}^{-1}\left(\frac{1}{{\lambda }_{2}}\gamma \right)\approx \frac{1}{{\lambda }_{2}}\gamma ,$$

$$\mathrm{sin}h\left(\frac{1}{{\lambda }_{2}}\gamma \right)\approx \frac{1}{{\lambda }_{2}}\gamma ,$$

5 $${\mathrm{tan}h}^{-1}\left(\frac{1}{{\lambda }_{2}}\gamma \right)\approx \frac{1}{{\lambda }_{2}}\gamma ,$$

The expressions of extra stress tensor given in Eq. (4) yield the following single form after using Eq. (5) in Eq. (4).

6 $${\varvec{S}}=\mu {\varvec{A}}+\frac{1}{{\lambda }_{1}{\lambda }_{2}}{\varvec{A}},$$

where $${\varvec{A}}=\nabla {\varvec{V}}+{\left(\nabla {\varvec{V}}\right)}^{t}$$.

For the ongoing problem, the direction of the time-dependent flow is taken towards the x-axis. So, we have the following form of velocity elements.

7 $${\varvec{V}}=\left(U\left(Y,t\right), 0, 0\right),$$

After using Eq. (7) in Eqs. (1) and (6) and Boussinesq’s approximation, the ongoing problem has the following form of momentum and energy equations^1,25,26^

8 $$\frac{\partial U\left(Y,t\right)}{\partial t}=\frac{1}{\rho }\left(\mu +\frac{1}{{\lambda }_{1}{\lambda }_{2}}\right)\frac{{\partial }^{2}U\left(Y,t\right)}{\partial {Y}^{2}}+g{\beta }_{T}\left(T\left(Y,t\right)-{T}_{\infty }\right),$$

9 $$\frac{\partial T\left(Y,t\right)}{\partial t}=\frac{k}{\rho {c}_{p}}\frac{{\partial }^{2}T\left(Y,t\right)}{\partial {Y}^{2}}-\frac{1}{\rho {c}_{p}}\frac{\partial {q}_{r}}{\partial Y},$$

With the Rosseland approximation of radiative heat flux ${q}_{r}=-\frac{4{\sigma }^{*}}{3{k}_{1}}\frac{\partial {T}^{4}}{\partial Y}$, Eq. (9) has the following expression^26^

10 $$\frac{\partial T\left(Y,t\right)}{\partial t}=\frac{1}{\rho {c}_{p}}\left(k+\frac{16{\sigma }^{*}{T}_{\infty }^{3}}{3{k}_{1}}\right)\frac{{\partial }^{2}T\left(Y,t\right)}{\partial {Y}^{2}},$$

The physical boundary conditions of the concerned problem are described as^27^

$$T\left(Y,t\right)={T}_{\infty }, U\left(Y,t\right)=0, Y>0, t=0,$$

$$T\left(Y,t\right)={T}_{w}, U\left(Y,t\right)=0, Y=0, t>0,$$

11 $$T\left(Y,t\right)={T}_{\infty }, U\left(Y,t\right)=0, Y\to \infty , t>0.$$

Now, familiarize the following non-dimensional variables^27^

12 $${T}^{*}\left( {Y}^{*},{t}^{*}\right)=\left(T\left(Y,t\right)-{T}_{\infty }\right)/{(T}_{w}-{T}_{\infty }), {t}^{*}={U}_{0}^{2}t/\upsilon , {U}^{*}\left({Y}^{*},{t}^{*}\right)=U\left(Y,t\right)/{U}_{0}, {Y}^{*}={U}_{0}Y/\upsilon ,$$

Equations (8) and (10) take the following form after the implementation of Eq. (12).

13 $$\frac{\partial {U}^{*}\left({Y}^{*},{t}^{*}\right)}{\partial {t}^{*}}=\left(1+N\right)\frac{{\partial }^{2}{U}^{*}\left({Y}^{*},{t}^{*}\right)}{\partial { {Y}^{*}}^{2}}+Gr{T}^{*}\left( {Y}^{*},{t}^{*}\right),$$

14 $$\frac{\partial {T}^{*}\left( {Y}^{*},{t}^{*}\right)}{\partial {t}^{*}}=\frac{1}{{Pr}_{eff}}\frac{{\partial }^{2}{T}^{*}\left( {Y}^{*},{t}^{*}\right)}{\partial {{Y}^{*}}^{2}},$$

The dimensionless form of Eq. (11) has the following expressions

$${T}^{*}\left({Y}^{*},{t}^{*}\right)=0, {U}^{*}\left( {Y}^{*},{t}^{*}\right)=0, {Y}^{*}>0, {t}^{*}=0,$$

$${T}^{*}\left({Y}^{*},{t}^{*}\right)=1, {U}^{*}\left( {Y}^{*},{t}^{*}\right)=0, {Y}^{*}=0, {t}^{*}>0,$$

15 $${T}^{*}\left({Y}^{*},{t}^{*}\right)=0, { U}^{*}\left( {Y}^{*},{t}^{*}\right)=0, {Y}^{*}\to \infty , {t}^{*}>0.$$

Here

16 $$N=\frac{1}{\mu {\lambda }_{1}{\lambda }_{2}}, Gr=\frac{g{\beta }_{T}\upsilon ({T}_{w}-{T}_{\infty })}{{U}_{0}^{3}}, Rd=\frac{16{\sigma }^{*}{T}_{\infty }^{3}}{3{k}_{1}k}, {Pr}_{eff}=\frac{Pr}{Rd+1}, Pr=\frac{\upsilon }{\alpha }.$$

### Fractional model

We implement the Caputo-Fabrizio time fractional derivative of order $\alpha \in \left[\mathrm{0,1}\right]$ to get the fractional model of the dimensionless system (13–14). In this fractional model, the time derivative is swapped with the Caputo-Fabrizio time fractional derivative which has the following form^28^

17 $${D}_{{t}^{*}}^{\alpha }{U}^{*}\left({Y}^{*},{t}^{*}\right)=\frac{1}{1-\alpha }{\int }_{0}^{{t}^{*}}Exp\left(\frac{\alpha ({t}^{*}-\xi }{\alpha -1}\right){{U}^{*}}{\prime}\left({Y}^{*},\xi \right)d\xi ,$$

In view of Eq. (17), Eqs. (13) and (14) take the following form

18 $${D}_{{t}^{*}}^{\alpha }{U}^{*}\left({Y}^{*},{t}^{*}\right)=\left(1+N\right)\frac{{\partial }^{2}{U}^{*}\left({Y}^{*},{t}^{*}\right)}{\partial {{Y}^{*}}^{2}}+Gr{T}^{*}\left( {Y}^{*},{t}^{*}\right)=0,$$

19 $${D}_{{t}^{*}}^{\alpha }{T}^{*}\left( {Y}^{*},{t}^{*}\right)=\frac{1}{{Pr}_{eff}}\frac{{\partial }^{2}{T}^{*}\left( {Y}^{*},{t}^{*}\right)}{\partial {{Y}^{*}}^{2}}.$$

### Exact solutions

To acquire the exact outcomes of the above Eqs. (18) and (19) with fractional derivative, we utilized the technique of Laplace transform. The powerful methodology of Laplace transform is utilized to examine the transient linear systems. This methodology is basically consistent for linear systems. With the help of this technique, various linear models used in physical systems can be effectively handled. The initial conditions through this methodology can be accurately incorporated into the analysis. The stability analysis can be accomplished in the engineering control system via this method. Firstly, we execute the Laplace transform method on Eq. (19) and then obtain the solution of Eq. (18). The Laplace transforms of velocity, temperature, and Caputo-Fabrizio time fractional derivative are defined in the following equation^28^

$$\overline{{U }^{*}}\left({Y}^{*},s\right)=\mathcal{L}\left[{U}^{*}\left({Y}^{*},{t}^{*}\right)\right]={\int }_{0}^{\infty }{U}^{*}\left({Y}^{*},{t}^{*}\right){e}^{-s{t}^{*}}d{t}^{*},$$

$$\overline{{T }^{*}}\left({Y}^{*},s\right)=\mathcal{L}\left[{T}^{*}\left({Y}^{*},{t}^{*}\right)\right]={\int }_{0}^{\infty }{T}^{*}\left({Y}^{*},{t}^{*}\right){e}^{-s{t}^{*}}d{t}^{*},$$

20 $$ {\mathcal{L}}\left[ {D_{{t^{*} }}^{\alpha } U^{*} \left( {Y^{*} ,t^{*} } \right)} \right] = \frac{{s{\mathcal{L}}\left[ {U^{*} \left( {Y^{*} ,t^{*} } \right)} \right] - U^{*} \left( {Y^{*} ,0} \right)}}{{\alpha + \left( {1 - \alpha } \right)s}}, $$

By using Eq. (20), Eq. (19) has the following form

21 $$\frac{{d}^{2}\overline{{T }^{*}}}{d{{Y}^{*}}^{2}}-{Pr}_{eff}\frac{s\gamma }{s+\alpha \gamma }\overline{{T }^{*}}=0,$$

where $$\gamma =\frac{1}{1-\alpha }.$$

The transformed boundary conditions of temperature are given as

22 $$\overline{{T }^{*}}\left(0,s\right)=\frac{1}{s}, \overline{{T }^{*}}\left(\infty ,s\right)=0.$$

Equation (21) has the following solution corresponding to Eq. (22)

23 $$\overline{{T }^{*}}\left({Y}^{*},s\right)=Exp\left(\sqrt{\frac{{Pr}_{eff}s\gamma }{s+\alpha \gamma }}{Y}^{*}\right){c}_{1}+Exp\left(-\sqrt{\frac{{Pr}_{eff}s\gamma }{s+\alpha \gamma }}{Y}^{*}\right){c}_{2},$$

where $${c}_{1}=0$$ and $${c}_{2}=\frac{1}{s}.$$

Another form of Eq. (23) can be written as follow

24 $$\overline{{T }^{*}}\left({Y}^{*},s\right)=\psi \left({Y}^{*}, s,{Pr}_{eff}\gamma ,\alpha \gamma \right),$$

As the inverse Laplace transform of the function ${\psi }_{1}\left(y, s,g,h\right)=\frac{1}{s}\mathrm{Exp}\left(-\sqrt{\frac{gs}{s+h}}y\right)$ is^28^

25 $${\psi }_{2}\left(y,t,g,h\right)={L}^{-1}{\psi }_{1}\left(y, s,g,h\right)=1-\frac{2g}{\pi }{\int }_{0}^{\infty }\frac{\mathrm{sin}\left(yx\right)}{x\left(g+{x}^{2}\right)}\mathrm{Exp}\left(\frac{-ht{x}^{2}}{g+{x}^{2}}\right)dx,$$

According to Eq. (25), the inverse Laplace transform of Eq. (24) has the following expression,

26 $${T}^{*}\left({Y}^{*},{t}^{*}\right)=1-\frac{2{Pr}_{eff}\gamma }{\pi }{\int }_{0}^{\infty }\frac{\mathrm{sin}\left({Y}^{*}x\right)}{x\left({Pr}_{eff}\gamma +{x}^{2}\right)}Exp\left(\frac{-\alpha \gamma {t}^{*}{x}^{2}}{{Pr}_{eff}\gamma +{x}^{2}}\right)dx,$$

To evaluate the above integral, we use the series representation of an exponential function. So, we have

27 $${\int }_{0}^{\infty }\frac{\mathrm{sin}\left({Y}^{*}x\right)}{x\left({Pr}_{eff}\gamma +{x}^{2}\right)}Exp\left(\frac{-\alpha \gamma {t}^{*}{x}^{2}}{{Pr}_{eff}\gamma +{x}^{2}}\right)dx=\sum_{n=0}^{\infty }\frac{{(-1)}^{n}{({t}^{*}\alpha \gamma )}^{n}}{n!}{\int }_{0}^{\infty }\frac{\mathrm{sin}\left({Y}^{*}x\right)}{x}{\left({Pr}_{eff}\gamma +{x}^{2}\right)}^{-1-n}({{x}^{2})}^{n}dx,$$

After evaluating the above integral, Eq. (27) can be written as

28 $$ \begin{aligned} & {\int }_{0}^{\infty }\frac{\mathrm{sin}\left({Y}^{*}x\right)}{x\left({Pr}_{eff}\gamma +{x}^{2}\right)}Exp\left(\frac{-\alpha \gamma {t}^{*}{x}^{2}}{{Pr}_{eff}\gamma +{x}^{2}}\right)dx\\ &\quad=\sum_{n=0}^{\infty }\frac{{(-1)}^{n}{({t}^{*}\alpha \gamma )}^{n}}{n!}\left[\frac{1}{4\Gamma \left[1+n\right]}{\left(\frac{1}{{Pr}_{eff}\gamma }\right)}^{-n}{\left({Pr}_{eff}\gamma \right)}^{-n}\right. \\ & \qquad \left.\left\{2\sqrt{\frac{1}{{Pr}_{eff}\gamma }}\sqrt{\pi }{Y}^{*}\Gamma \left[\frac{1}{2}+n\right]\mathrm{ HypergeometricPFQ}  \left[\left\{\frac{1}{2}+n\right\}, \left\{\frac{1}{2},\frac{3}{2}\right\}, \frac{{Pr}_{eff}\gamma {{Y}^{*}}^{2}}{4} \right]\right.\right.  \\  & \qquad \left. \left. -\pi {Y}^{*}\sqrt{{{Y}^{*}}^{2}}\Gamma \left[1+n\right]\mathrm{HypergeometricPFQ}\left[\left\{1+\mathrm{n}\right\}, \left\{\frac{3}{2},2\right\},\frac{{Pr}_{eff}\gamma {{Y}^{*}}^{2}}{4}\right]\right\} \right],\end{aligned} $$

We get the exact solution of the temperature distribution after using Eqs. (27) and (28) as follows

29 $$ \begin{aligned} & {T}^{*}\left({Y}^{*},{t}^{*}\right)=1-\frac{2{Pr}_{eff}\gamma }{\pi }\sum_{n=0}^{\infty }\frac{{\left(-1\right)}^{n}{\left({t}^{*}\alpha \gamma \right)}^{n}}{n!}\\ &\quad\left[\frac{1}{4\Gamma \left[1+n\right]}{\left(\frac{1}{{Pr}_{eff}\gamma }\right)}^{-n}{\left({Pr}_{eff}\gamma \right)}^{-n}\left\{2\sqrt{\frac{1}{{Pr}_{eff}\gamma }}\sqrt{\pi }{Y}^{*}\Gamma \left[\frac{1}{2}+n\right]\mathrm{ HypergeometricPFQ} \right.\right.\\ &\quad\left.\left.\left[\left\{\frac{1}{2}+n\right\}, \left\{\frac{1}{2},\frac{3}{2}\right\}, \frac{{Pr}_{eff}\gamma {{Y}^{*}}^{2}}{4} \right]-\pi {Y}^{*}\sqrt{{{Y}^{*}}^{2}}\Gamma \left[1+n\right]\mathrm{HypergeometricPFQ}\right.\right.\\ &\quad\left.\left.\left[\left\{1+\mathrm{n}\right\}, \left\{\frac{3}{2},2\right\},\frac{{Pr}_{eff}\gamma {{Y}^{*}}^{2}}{4}\right]\right\} \right].\end{aligned} $$

Now to acquire the solution of the velocity distribution given in Eq. (18), we apply the Laplace transform technique and get the following form

30 $$\left(1+N\right)\frac{{d}^{2}\overline{{U }^{*}}}{d{{Y}^{*}}^{2}}-\frac{s\gamma }{s+\alpha \gamma }\overline{{U }^{*}}+Gr\overline{{T }^{*}}=0,$$

The transformed conditions are

31 $$\overline{{U }^{*}}\left(0,s\right)=0, \overline{{U }^{*}}\left(\infty ,s\right)=0.$$

Equation (30) has the following solution

32 $$ \begin{aligned}\overline{{U }^{*}}\left({Y}^{*},s\right)&=\frac{Gr(s+\alpha \gamma )}{{s}^{2}\left(\gamma -\left(1+N\right){Pr}_{eff}\gamma \right)} Exp\left(-\sqrt{\frac{{Pr}_{eff}s\gamma }{(s+\alpha \gamma )}}{Y}^{*}\right)\\ &\quad+{c}_{3}Exp\left(\sqrt{\frac{s\gamma }{(1+N)(s+\alpha \gamma )}}{Y}^{*}\right){+c}_{4}Exp\left(-\sqrt{\frac{s\gamma }{(1+N)(s+\alpha \gamma )}}{Y}^{*}\right)\end{aligned} $$

Using Eq. (31), we have the values of constants ${c}_{3}=0, {c}_{4}=-\frac{Gr(s+\alpha \gamma )}{{s}^{2}\left(\gamma -\left(1+N\right){Pr}_{eff}\gamma \right)}.$

Equation (32) can be written as

33 $$\overline{{U }^{*}}\left({Y}^{*},s\right)=\frac{Gr(s+\alpha \gamma )}{{s}^{2}\left(\gamma -\left(1+N\right){Pr}_{eff}\gamma \right)} Exp\left(-\sqrt{\frac{{Pr}_{eff}s\gamma }{(s+\alpha \gamma )}}{Y}^{*}\right)-\frac{Gr\left(s+\alpha \gamma \right)}{{s}^{2}\left(\gamma -\left(1+N\right){Pr}_{eff}\gamma \right)}Exp\left(-\sqrt{\frac{s\gamma }{\left(1+N\right)\left(s+\alpha \gamma \right)}}{Y}^{*}\right),$$

Equation (33) can also be written as follows

34 $$ \begin{aligned}\overline{{U }^{*}}\left({Y}^{*},s\right)&=\frac{{\beta }_{0}}{s} Exp\left(-\sqrt{\frac{{\delta }_{0}s}{({\delta }_{1}+s)}}{Y}^{*}\right)+\frac{{\beta }_{1}}{{s}^{2}} Exp\left(-\sqrt{\frac{{\delta }_{0}s}{({\delta }_{1}+s)}}{Y}^{*}\right)\\ &\quad-\frac{{\beta }_{0}}{s} Exp\left(-\sqrt{\frac{{\delta }_{2}s}{\left({\delta }_{1}+s\right)}}{Y}^{*}\right)- \frac{{\beta }_{1}}{{s}^{2}} Exp\left(-\sqrt{\frac{{\delta }_{2}s}{\left({\delta }_{1}+s\right)}}{Y}^{*}\right),\end{aligned} $$

where

$${\beta }_{0}=\frac{Gr}{\left(\gamma -\left(1+N\right){Pr}_{eff}\gamma \right)}, {\beta }_{1}=\frac{Gr\alpha \gamma }{\left(\gamma -\left(1+N\right){Pr}_{eff}\gamma \right)}, {\delta }_{0}={Pr}_{eff}\gamma , {\delta }_{1}=\alpha \gamma ,$$

35 $${\delta }_{2}=\frac{\gamma }{(1+N)}.$$

The more suitable expression of Eq. (34) can be written as follows,

36 $$\overline{{U }^{*}}\left({Y}^{*},s\right)={\beta }_{0}{\overline{\varphi } }_{1}\left({Y}^{*},s,{\delta }_{0},{\delta }_{1}\right)+{\beta }_{1}{\overline{\varphi } }_{2}\left({Y}^{*},s,{\delta }_{0},{\delta }_{1}\right)-{\beta }_{0}{\overline{\varphi } }_{3}\left({Y}^{*},s,{\delta }_{1},{\delta }_{2}\right)-{\beta }_{1}{\overline{\varphi } }_{4}\left({Y}^{*},s,{\delta }_{1},{\delta }_{2}\right).$$

The solution of velocity distribution is achieved after applying the inverse Laplace transformation on Eq. (36) as follows,

37 $${U}^{*}\left({Y}^{*},{t}^{*}\right)={\beta }_{0}{\varphi }_{1}({Y}^{*},{t}^{*},{\delta }_{0},{\delta }_{1})+{\beta }_{1}{\varphi }_{2}({Y}^{*},{t}^{*},{\delta }_{0},{\delta }_{1})-{\beta }_{0}{\varphi }_{3}({Y}^{*},{t}^{*},{\delta }_{1},{\delta }_{2})-{\beta }_{1}{\varphi }_{4}({Y}^{*},{t}^{*},{\delta }_{1},{\delta }_{2})$$

where ${\varphi }_{1},{\varphi }_{2},{\varphi }_{3}, {\varphi }_{4}$ can be evaluated in a same manner as ${\psi }_{2}(y, t,g,h)$ in Eq. (25).

## Analysis and discussion of results

### Analysis of results

The fractional time derivative study of Nadeem trigonometric non-Newtonian fluid with an unsteady radiative flow over a vertical infinite plate has been examined in this article. The execution of adequate variables on the constitutive equations of the problem yields the dimensionless setup of equations. To get more significant results of the flow phenomenon, the fractional model of the Caputo-Fabrizio time derivative is implemented on the dimensionless system of coupled equations. The exact solutions of the temperature distribution and velocity distribution are obtained through an effective methodology of the Laplace transformation. For the correctness and confirmation of the ongoing problem, the temperature distribution values for specific parameters are compared with the previous study in Table 1. These values exhibit an excellent bonding with the previous findings, which indicate the validation of the present analysis. The physical interpretation of temperature and velocity fields relative to the pertinent parameters and time are graphically explored. The flow and thermal distributions of the considered fluid regarding different time periods and physical parameters are physically visualized. From the graphical representation of the temperature distribution and velocity distribution, it is perceived that the graphical results are more significant in the context of a larger period of time as compared to a smaller period of time.

**Table 1:** Comparison of the values of the temperature distribution with Fetecau et al.^32^ for ${t}^{*}=0.5$

$$\alpha =0.1$$		
${Y}^{*}$	Present findings	Fetecau et al. ^32^
0.1	0.86264	0.86341
0.2	0.74408	0.74363
0.3	0.64175	0.64057
0.4	0.55344	0.55187
0.5	0.47724	0.47551

### Discussion of results

**Figure 2:**
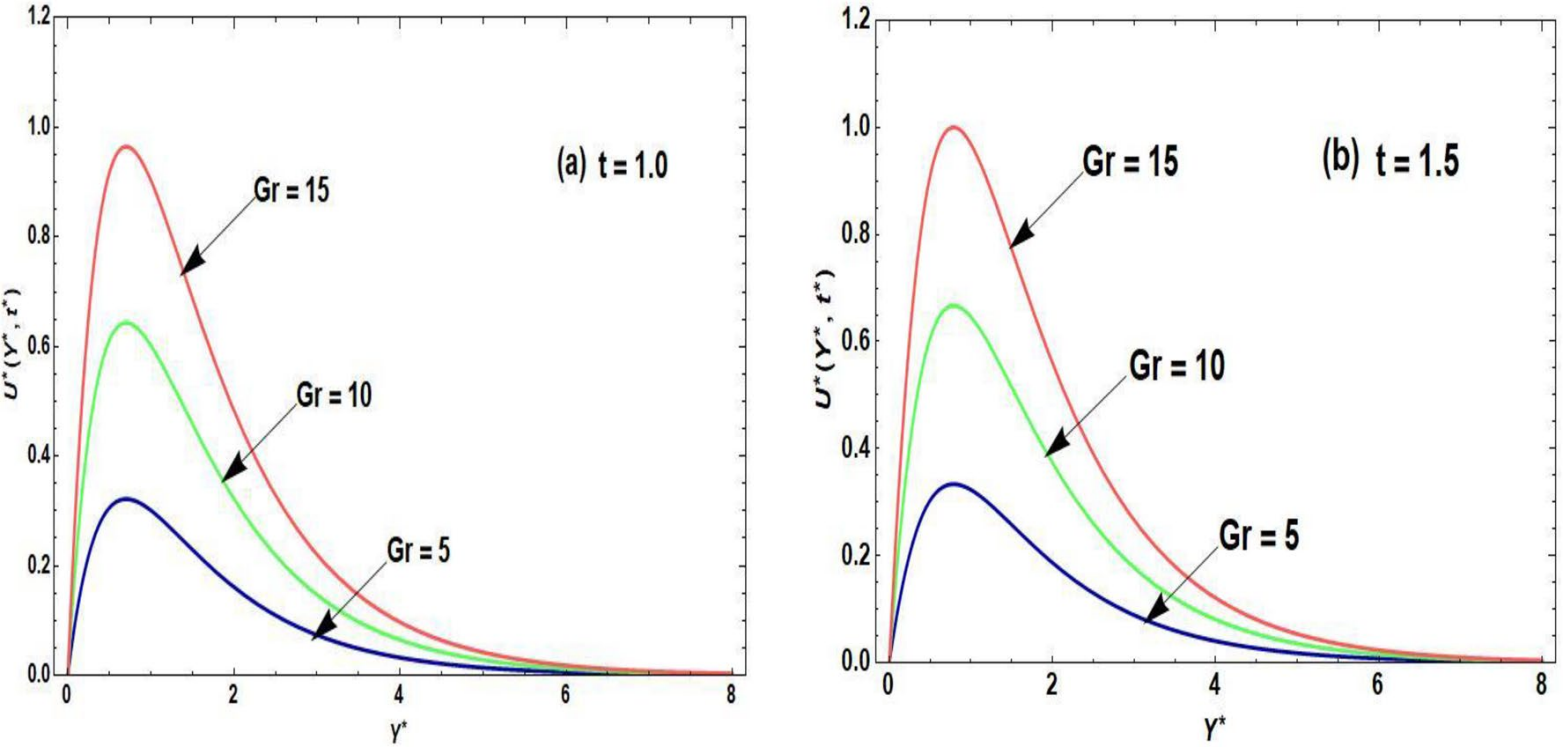
(**a**,**b**) Velocity distribution regarding Grashof number and time.

**Figure 3:**
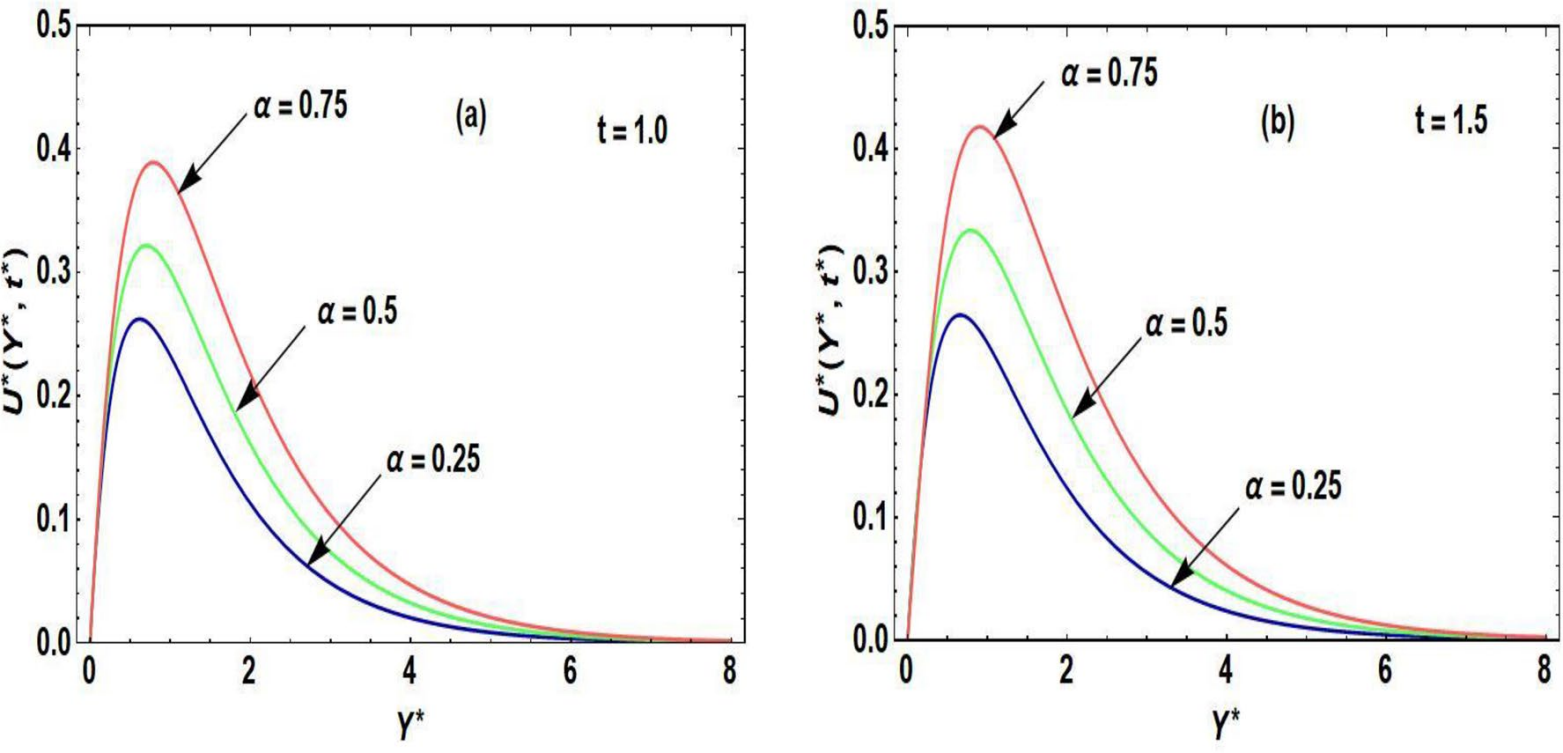
(**a**,**b**) Velocity distribution regarding fractional parameter and time.

**Figure 4:**
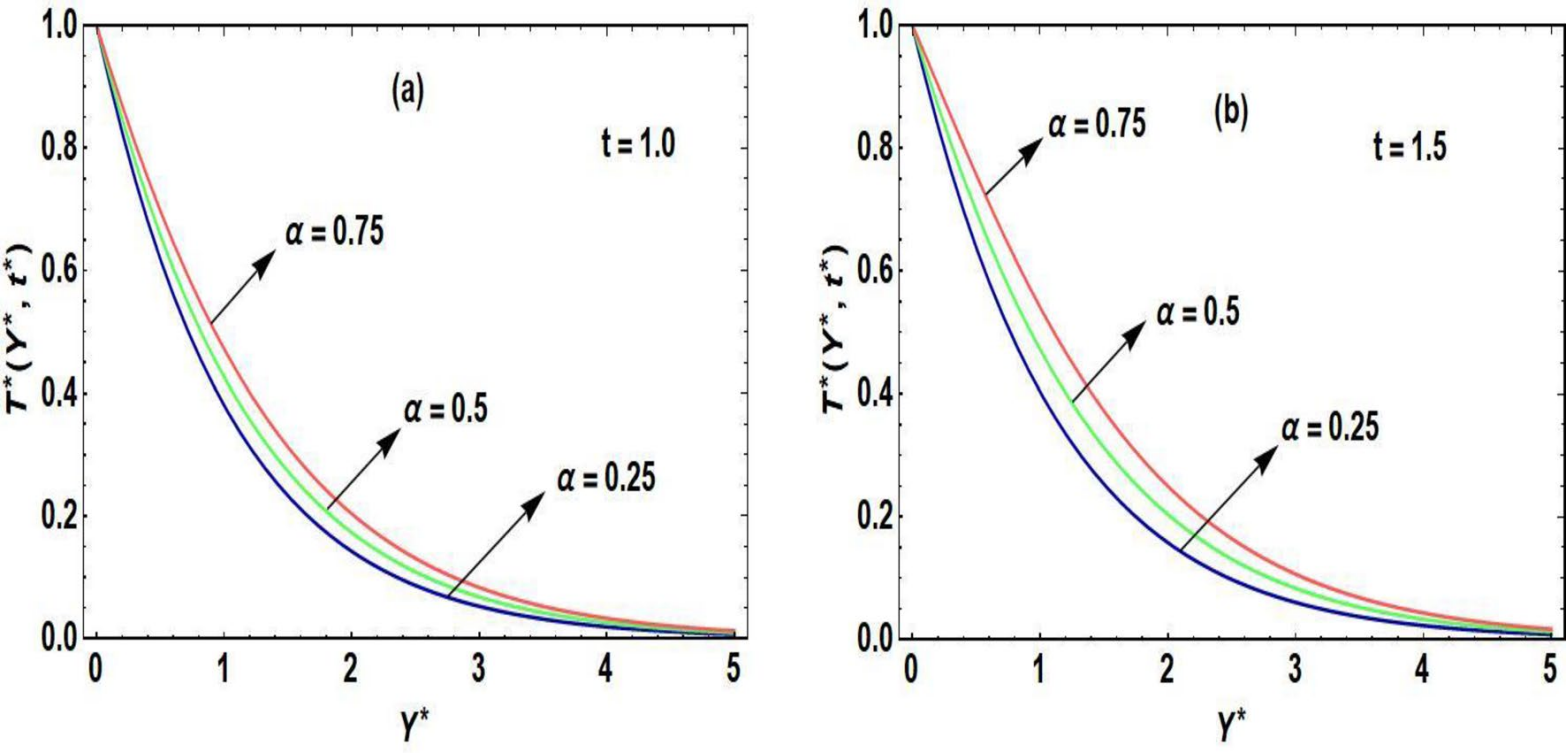
(**a**,**b**) Temperature distribution regarding fractional parameter and time.

**Figure 5:**
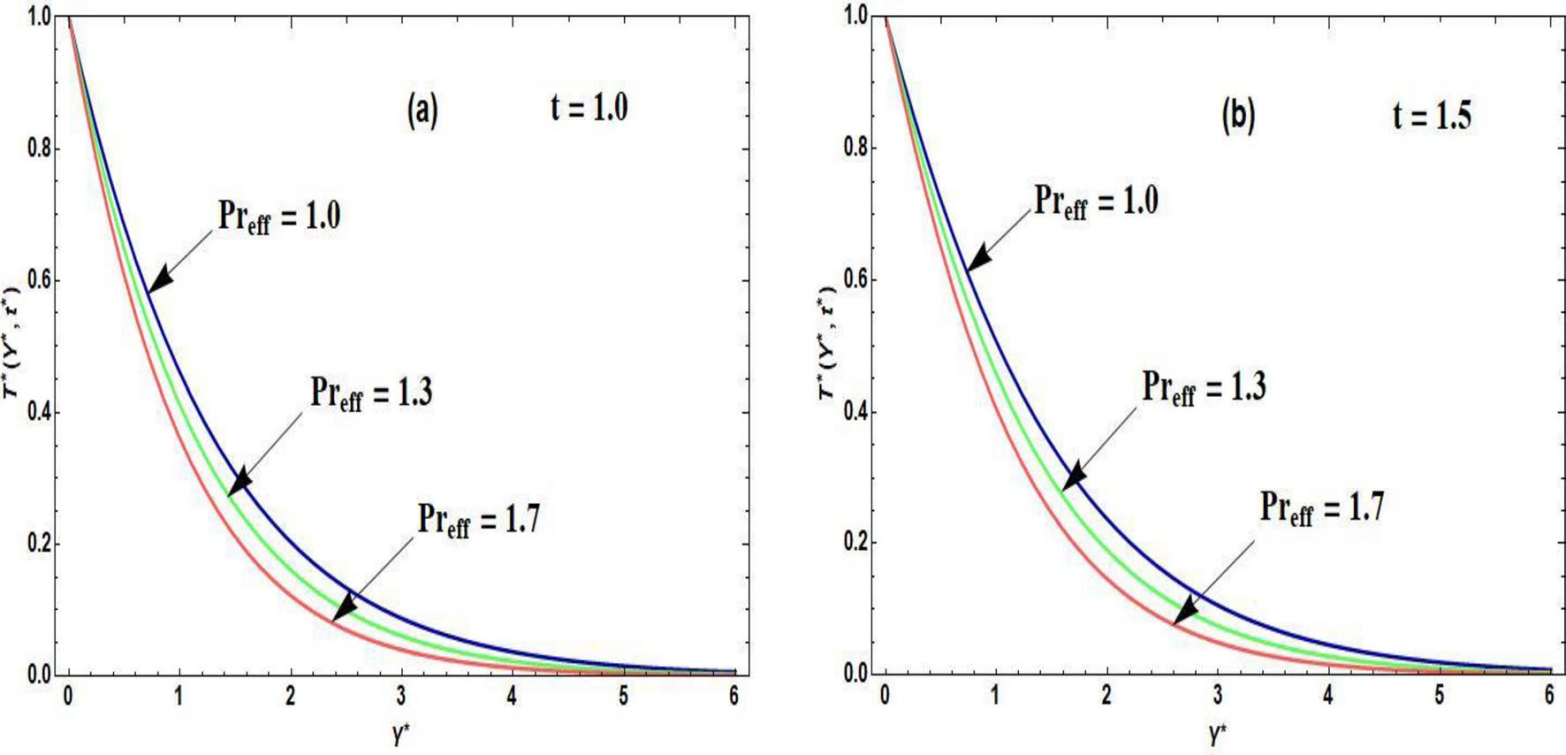
(**a**,**b**) Temperature distribution regarding effective Prandtl number and time.

Figures 2a,b–5a,b are prepared to examine the influence of the pertinent parameters on the velocity field and temperature field regarding distinct periods of time. The purpose of organizing Fig. 2a,b is to disclose the nature of the velocity curve relative to the distinct time periods and escalation of the Grashof number. The increment of the Grashof number for both intervals of time develop an augmentation in the field of velocity. The reason for this graphical phenomenon is that the Grashof number characterized the connection of buoyancy and restraining forces. The reason for buoyancy forces is the variation of the fluid density across space. On the other hand, the viscosity of the fluid is the cause of the restraining forces. According to Shah et al.^29^, an increase in buoyancy results in a higher velocity of fluid flow, as described by Archimedes’ principle. This principle exhibits that the buoyant force operating on an object submerged in a fluid is equivalent to the displaced fluid’s weight. In a related study, Animasaun et al.^30^ identified that when an object in a fluid experiences a greater buoyant force, it effectively reduces the net force acting against its motion, leading to an increase in velocity. Accordingly, the fluid accelerates objects with enhanced buoyancy more rapidly, resulting in a corresponding acceleration in the fluid velocity. This principle executes practical implementation in fields such as fluid dynamics, where the movement of objects in fluids is examined. Moreover, it plays a role in natural phenomena like the rising of hot air in a less dense and buoyant manner, contributing to atmospheric circulation patterns^31^. Figure 3a,b is sketched to observe the accelerating order of the fractional derivative at two different times on the curve of the fluid velocity. The larger magnitude of the fractional derivative yields an acceleration in the field of velocity. In Fig. 3b, at the large time period, the increasing effects of the fractional parameter are dominating near the plate as compared to the small period of time in Fig. 3a. According to the physical point of view, the thickness of the boundary layer becomes augmented due to the increment of fractional derivative order. Consequently, the flow field exhibits accelerating behavior. At both small and large time periods, Fig. 4a,b depicts the nature of temperature distribution corresponding to the escalating order of fractional derivative. The profile of the temperature is augmented with the increasing order of the fractional derivative for both time periods. The reason behind this phenomenon is that when the fractional operator’s order is enhanced, the memory effect confined in the fractional operator becomes augmented which further affects the time period. Accordingly, with the increasing fractional parameter or fractional operator’s order, the period of time is enhanced which escalates the temperature profile. The significance of the higher values of the effective Prandtl number relative to the different time periods on the field of the temperature is shown in Fig. 5a,b. The temperature distribution declines due to the accelerating magnitude of the effective Prandtl number. As the Prandtl number is inversely related to the fluid’s thermal diffusivity. The thermal diffusivity becomes declines due to the improved Prandtl number which means that the rate of heat transfer through the fluid is lower. Consequently, the temperature distribution shows a declining behavior.

## Conclusion

The ongoing study focuses on the time-dependent flow phenomenon with the heat transfer mechanism in a trigonometric non-Newtonian fluid based on the fractional model. The fractional model of a trigonometric non-Newtonian fluid is obtained through the application of the Caputo-Fabrizio fractional derivative of order $\alpha$. The thermal analysis of the problem is described with the significance of the thermal radiation. The suitable similarity variables yield the dimensionless setup of the equations. The implementation of the Laplace transform technique on the dimensionless setup of the equations provides the exact outcomes. This study has the following essential outcomes.

- Both the temperature and velocity distributions have dominating impacts related to the pertinent parameters at the large time period in comparison to the small time period.
- The field of the temperature demonstrates the declining nature corresponding to the larger effective Prandtl number.
- At both small and larger time periods, the purpose of the improved fractional parameter is to enhance the temperature profile.
- The consequence of the fractional parameter on the nature of the velocity distribution is the same as in the case of temperature distribution. However, in a larger time period, the effects are significant.
- In both time periods, the flow velocity becomes escalating influenced by the greater intensity of the fluid parameter.
- During both small and large time intervals, the improved Grashof number exaggerates the velocity distribution.

## Abbreviations

*k*: Fluid thermal conductivity
${\lambda }_{1}{\lambda }_{2}$: Material constants of Eyring-Powell fluid
${\beta }_{T}$: Thermal expansion coefficient
$\boldsymbol{\Gamma }$: Extra stress tensor
${k}_{1}$: Mean absorption coefficient
$$\upsilon $$: Kinematic viscosity
*N*: Fluid parameter
${Pr}_{eff}$: Effective Prandtl number
*t*: Time
${T}_{\infty }$: Free stream temperature
*s*: Laplace transform parameter
***A***: First Rivlin -Erickson tensor
*U*: Velocity of the fluid
*g*: Gravitational acceleration
$\mu$: Dynamic fluid viscosity
${T}_{w}$: Temperature at the surface
***V***: Velocity vector
${\sigma }^{*}$: Stefan-Boltzmann constant
$Gr$: Thermal Grashof number
${c}_{p}$: Specific heat capacity
$\alpha$: Non-integer order of fractional operator
*Rd*: Radiation parameter
